# The Role of Th17 Cells and IL-17 in Th2 Immune Responses of Allergic Conjunctivitis

**DOI:** 10.1155/2020/6917185

**Published:** 2020-05-24

**Authors:** Xiang-Tian Meng, Yun-Yue Shi, Hong Zhang, Hong-Yan Zhou

**Affiliations:** ^1^Department of Ophthalmology, China-Japan Union Hospital of Jilin University, Changchun 130033, Jilin Province, China; ^2^Department of Obstetrics and Gynecology, China-Japan Union Hospital of Jilin University, Changchun 130033, Jilin Province, China

## Abstract

Allergic conjunctivitis (AC) is a common allergic disease that is often associated with the onset of rhinitis or asthma. The incidence of AC has increased significantly in recent years possibly due to air pollution and climate warming. AC seriously affects patients' quality of life and work efficiency. Th (T-helper) 2 immune responses and type I hypersensitivity reactions are generally considered the basis of occurrence of AC. It has been found that new subpopulations of T-helper cells, Th17 cells that produce interleukin-17 (IL-17), play an important role in the Th2-mediated pathogenesis of conjunctivitis. Studies have shown that Th17 cells are involved in a variety of immune inflammation, including psoriasis, rheumatoid arthritis, inflammatory bowel disease, systemic lupus erythematosus, and asthma. However, the role of Th17 and IL-17 in AC is unclear. This paper will focus on how T-helper 17 cells and interleukin-17 are activated in the Th2 immune response of allergic conjunctivitis and how they promote the Th2 immune response of AC.

## 1. Introduction

Allergic conjunctivitis (AC) is an inflammatory disorder of conjunctivae which negatively affects the family and daily activities and is responsible for significant work and school absenteeism [1, 2]. The prevalence can vary in intensity, seasonality, gender, country, and region [3–6]. However, the consensus is that a number of researchers have reported that the incidence of allergic diseases, including various types of allergic conjunctivitis, has increased significantly [7–10]. This discrepancy could be attributed to indoor and outdoor air pollution and the increased pollen due to climate change and global warming [11–14]. Several reports have shown that AC is closely related to asthma, rhinitis, and other allergic diseases, which seriously affects the quality of life of patients and productivity [5–7, 15–17]. In addition, the incidence of AC is related to many factors. The prevalence of allergic rhinitis, allergic conjunctivitis, and asthma has significantly increased among the general population, especially in developed cities with severe air pollution. This phenomenon supports the link between industrialization and allergic diseases [18]. Gabet et al. demonstrated that children who are highly sensitive to dust mites have the highest risk of developing allergic diseases [19]. The condition is often classified as seasonal allergic conjunctivitis (SAC), perennial allergic conjunctivitis (PAC), atopic keratoconjunctivitis (AKC), vernal keratoconjunctivitis (VKC), and giant papillary conjunctivitis (GPC) [1, 20, 21]. Th2 immune responses and type I hypersensitivity reactions are generally considered the basis of occurrence of AC [22]. An European Academy of Allergy and Clinical Immunology (EAACI) task force suggested to include “ocular allergy” in the “ocular surface hypersensitivity disorders,” dividing the different forms into IgE-mediated and non-IgE-mediated diseases [20, 23]. SAC and PAC are typical IgE-mediated allergic reactions. AKC and VKC include both IgE-mediated immunity and non-IgE-mediated immunity. GPC is a disease related to contact lenses wear, which is not considered any longer as an allergic disorder but still included within the allergic conjunctivitis. SAC and PAC do not have discernible difference in the symptoms such as ocular itching, hyperaemia, dry eye, redness, and lid swelling, and also, tearing, mucous discharge, and burning may occur [20, 24–26]. They all belong to the acute type of allergic conjunctivitis. However, SAC, due to airborne pollen allergens, usually occurs during allergy season in spring and summer [10, 27]. Patients sensitized to perennial allergens instead, like insects, household molds, house dust mites, or animal epithelia, can suffer from PAC and experience symptoms throughout the year [10, 20, 27]. So, the key differentiator between SAC and PAC is their occurrence time and duration of discomfort. Therefore, some scholars believe that SAC and PAC are actually the same disease manifested in different forms [28]. Since SAC and PAC as well as intermittent and persistent rhinitis often occur together, while eye or nasal symptoms alone are rare, they are grouped together as allergic rhinoconjunctivitis [2, 29]. In temperate zones, the SAC percentage is 90 percent, and the PAC percentage is 5 percent; however, in tropical climates, PAC seems to be more common [8, 16]. So, they have a significant impact on the quality of patient's life and affect social economy [30]. Although VKC and AKC account for only 2% of ocular allergy cases, they have a greater impact on life [22]. Unlike SAC and PAC, VKC and AKC appear as corneal involvement. So, AKC and VKC are sight-threatening keratoconjunctivitis [8, 31]. Infiltration and activation of eosinophils are the main causes of corneal complications in chronic allergic diseases [32]. It has been found that new subpopulations of T-helper (Th) cells, Th17 cells that produce interleukin-17 (IL-17), play an important role in the Th2-mediated pathogenesis of conjunctivitis. Studies have shown that Th-17 cells are involved in a variety of immune inflammation, including psoriasis, rheumatoid arthritis, inflammatory bowel disease, systemic lupus erythematosus, and asthma [33, 34]. However, the role of Th17 and IL-17 in AC is unclear. Therefore, this article will focus on the activation and action of Th17 cells in the Th2-type immune responses to introduce the immunological mechanism of AC and the new progress in diagnosis and treatment.

## 2. Classical Biological Mechanisms

**Figure 1:**
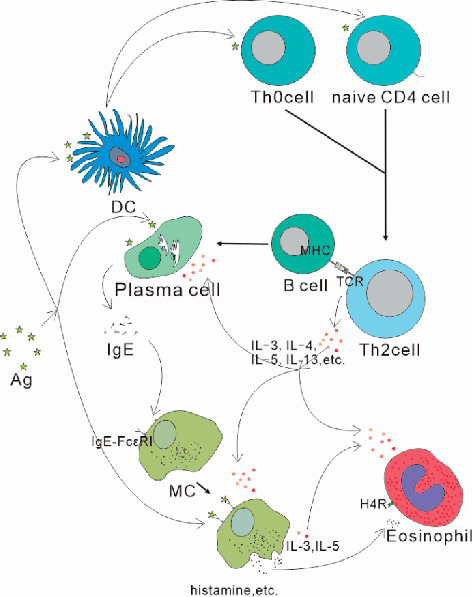
In classical type I hypersensitivity reactions, activation of individual cells and immune molecules finally results in mast cell degranulation and eosinophil infiltration. Factors such as IL-3, IL-4, IL-5, and IL-13 produced by Th2 cells promote this process.

Conjunctiva is one of the most common sites of allergic inflammation due to direct exposure of the conjunctiva and easy contact with allergens. In the sensitization phase, the initial genetic susceptibility of individual ocular exposure to a novel allergen, which is processed and presented by dendritic cells (DC) and/or other antigen-presenting cells (APC), causes naive CD4 cells or helper T cells (Th0) to mature and differentiate into Th2 lymphocytes [35]. Sensitization and differentiation of Th2 cells require antigen presentation by DC [35]. Th2 cells mainly participate in IgE-mediated allergies through the release of IL-3, IL-4, IL-5, IL-9, IL-10, and IL-13. These allergies include B cells producing IgE, mast cell growth, and aggregation of acid granulocytes [32]. When the antigen peptide-MHC molecule located on the surface of B cells interacts with the TCR located on the surface of CD4 cells, B cells proliferate and differentiate into plasma cells, secrete antigen-specific IgE, and aggregate the high-affinity IgE receptor (Fc*ε*RI) located on the surface of mast cells (MC) and basophils [32]. Th2-derived cytokines, such as IL-4 and IL-5, are involved in eosinophil activation and chemotaxis [36, 37]. When the same allergen is encountered as the eye was previously allergic, the allergen attaches to the IgE-Fc*ε*RI complex and cross-links to the mast cell surface. MCs express Fc*ε*RI, IgE-Fc*ε*RI complex, and allergen-based epitope cross-link to activate mast cells to release their preformed mediators such as histamine, proteolytic enzymes, and proteoglycans as part of the early response and then the reaction part as a late rapid synthesis of leukotrienes and prostaglandin lipid mediators [38]. IgE-Fc*ε*RI cross-linking generates a signal that lyses mast cell membrane phospholipids, releasing them, and produces a wide range of MC-derived mediators, including IL-2, IL-3, IL-4, IL-5, IL-6, IL-10, IL-12, granulocyte-macrophage colony-stimulating factor (GM-CSF), and TNF-*α* [39, 40]. Among them, IL-3 and IL-5 are involved in the development, survival, and recruitment of eosinophils, which are helpful for the occurrence of eosinophilic inflammation. Histamine is the main agent involved in ocular anaphylaxis [41]. Among the known histamine receptors, H1R, H2R, and H4R subtypes are closely related to eye allergies. Histamine signals have been shown to increase conjunctival hyperemia, fibroblast proliferation, cytokine secretion, expression of adhesion molecules, microvascular permeability, and procollagen production through H1R and H2R [41]. H4R regulates a variety of physiological functions, including the release of cytokines and chemokines, expression of adhesion molecules and chemotaxis, and recruitment of mast cells, eosinophils, dendritic cells, and lymphocytes into the conjunctiva [42, 43]. Then, in late-phase response, activated eosinophils result in the release of inflammatory cytokines including eosinophil cationic protein (ECP), eosinophil peroxidase, neutrophil toxic oxygen free radicals, proteases, and Th2 lymphocytes, among others, some such as major basic proteins (MBP) and ECP are toxic to the corneal epithelium [44, 45] (Figure 1).

## 3. Th17 and IL-17 in AC

### 3.1. Th17 and IL-7 May Promote AC

Th17 is a T-cell lineage different from Th1 and Th2 cells and is considered to be a novel preinflammatory T effector cell [46]. In 2005, researchers found so-called helper T cells, “Th17 subsets,” in mice as a T helper subset distinct from Th1 and Th2 cells [47]. It is mainly in the regulation of immune responses and clearance of extracellular pathogens that TH17 cells play a role [48]. Retinoid-related orphan receptor *γ*t (ROR*γ*t) is needed in Th17 cell differentiation [49]. IL-17A (also called IL-17) is the signature cytokine of Th17 cells [48], but they also produce IL-17F, IL-22, and GM-CSF [50–52]. Among them, IL-17A also is the most widely distributed [53]. Although IL-17 is most richly expressed by Th17 cells, it can also be produced by other immune cells, including macrophages, B cells, natural killer T cells, innate lymphocytes, and CD8+T cells [54]. Indeed, IL-17 and Th17 cells have been shown to be associated with human autoimmune diseases in many tissue regions, including psoriasis, rheumatoid arthritis, inflammatory bowel disease, systemic lupus erythematosus, and asthma [55–61]. Recent evidence suggests that Th17 cells are also associated with Th2 hypersensitivity [62, 63]. Extensive data indicate that Th17 cells and IL-17 have proinflammatory roles in allergic airway disease [64]. The concentrations of IL-17A, IL-17F, and IL-22 in bronchoalveolar lavage fluid and bronchi in asthmatic patients are positively correlated with disease severity and airway responsiveness [65, 66]. This further aggravates the allergic reaction [67]. IL-17-deficient mice reduced airway inflammation after Ag attack [68], and neutralization of IL-17 reduced Th2-induced allergic airway disease [55, 69, 70]. Recently, it reported that ROR*γ*t and IL-17 levels were elevated in nasal lavage fluid in allergic rhinitis mice [71]. Kinyanjui et al.'s data also show that low doses of IL-17 can enhance Th2-dependent airway inflammation [64]. Castaneda et al.'s experiments also support the hypothesis that air pollution exacerbates the allergic immune response by enhancing the Th17 immune response [72]. Currently, Th17 cells or IL-17 have also been found in inflammatory diseases of the eye such as uveitis, scleritis, diabetic retinopathy, and dry eye [73–77]. All these suggest that both Th17 cells and IL-17 may participate in and deepen the Th2 response in AC [73]. The role of Th17 cells in allergic conjunctivitis is a relatively new concept. However, the role of Th17 in AC is largely unknown. In a recent experiment, the significant stimulation and activation of Th17 cytokines, IL-17A and IL-17F, and the specific transcription factor ROR*γ*t in a mouse model of allergic conjunctivitis showed when developmental enhancement can aggravate Th2-dominant allergic inflammation in allergic eye disease [78].

### 3.2. Activation of Th17 Cells

**Figure 2:**
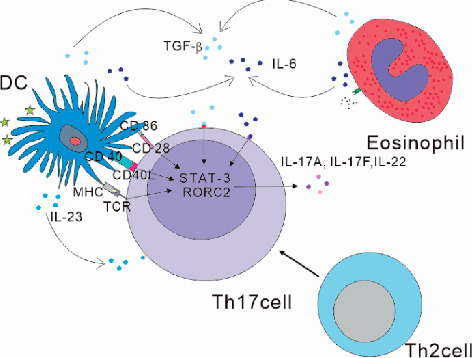
1. CD40 and CD86 signaling on DC appears to be critical in the induction of Th17 cells. 2. IL-23 supports the maintenance of Th17 cell function. 3. IL-6 and TGF-*β* produced by eosinophil and DC expression promote Th17 cell differentiation. 4. Activated Th2 cells can be directly transformed into Th17 cells.

Activation state of DC is essential to Th2 cells and Th17 cells [79, 80]. DC are “important APC” and play an important role in presenting antigens and inducing primary immune responses. Kudo M et al.'s studies suggest that Th17 cell differentiation may be associated with avb8 integrin on DCs [81]. CD40 and CD86 signaling appears to be critical in the induction of Th17 cells [82]. In the meantime, because differentiated TH cells have plasticity, Th2 cells can be differentiated into Th2/Th17 cells [83]. This means that, in eye allergies, activated Th2 cells can be directly transformed into Th17 cells. Meanwhile, when natural T cells are activated under the action of transforming growth factor *β* (TGF-*β*) and IL-6 and IL-23 secreted by APC, signal transduction and activation of transcription factor 3 (STAT3) and RORC2 are activated to differentiate into Th17 cells [82, 84]. And then, IL-23 will support the maintenance of Th17 cell function [85]. Furthermore, expression of IL-17A has been reported to be related to eosinophils which produced IL-6 and TGF-*β* [48, 53, 86]. So, DC and eosinophils activated in AC may support Th17 differentiation [62, 65, 79] (Figure 2).

### 3.3. Role of IL-17 in Th2 Immune Responses

**Figure 3:**
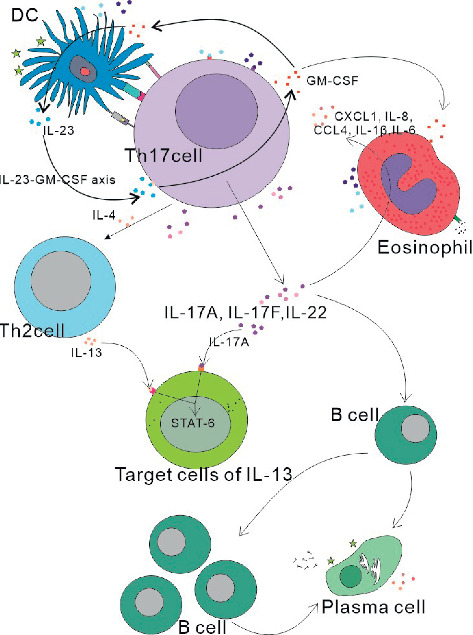
1. The immune response is maintained by the IL-23-GM-CSF axis. 2. IL-17A and IL-17F can promote the production of eosinophils CXCL1, IL-8, and CCL4, as well as IL-1*β* and IL-6. 3. IL-17A is involved in the pathophysiology of allergies by increasing the ability of IL-13 to activate signaling pathways such as intracellular STAT-6. IL-17A protein expression synergizes with IL-13. 4. Th17 cells have been shown to help B cell differentiation and to play a key role in the formation of ectopic lymphoid follicles in the target organ.

IL-17 is a well-known proinflammatory property. IL-17 plays an important role in maintaining health in response to injury, physiological stress, and infection [60]. The exact role of IL-17 in AC is unclear. Interestingly, it proved to be not only a positive role in regulating the immune response but also a negative regulatory role as well [87]. Some studies have shown that Th17 cell development is enhanced, which exacerbates the dominance of Th2 [73]. Due to the plasticity of differentiated T helper cells, under the stimulation of IL-4, Th17 cells can be transformed into IL-2 producing Th2 cells [85]. Adoptive transfer of Th17 cells and Th2 cells can promote antigen-induced Th2-mediated eosinophil inflammation [63]. T cells that produce IL-17 induce neutrophilia in mice, and these cells also actively regulate Th2-driven eosinophilia [64]. Studies have found that the Th2 response is weakened in the absence of IL-17R signals due to impaired Th2 cell activation, and mouse models lacking the IL-17R gene show airway eosinophil recruitment, and eosinophil peroxidation activity was reduced [88]. Some scholars have confirmed that IL-17A and IL-17F can promote the production of eosinophils CXCL1, IL-8, and CCL4, as well as IL-1*β* and IL-6 [74, 78]. This may be related to the fact that the IL-17 signal promotes the interactions required to promote germinal center (GC) formation of CD4+T cells and B cells [89]. Meanwhile, the GC-B cell development and humoral responses of the mouse lacking the IL-17 receptor were reduced, which suggests a mechanism through which IL-17 drives the autoimmune response by promoting the formation of spontaneous GCs [89]. In addition to these, Th17 cells have been shown to help B cell differentiation and to play a key role in the formation of ectopic lymphoid follicles in the target organ [89]. Other researchers also demonstrated Th17 cells as helper B cells because they not only help in in vitro proliferation of B cells to produce a strong reaction but also class switch recombination in vivo by triggering the production of antibodies [90]. Eosinophils are derived from progenitor cells in the bone marrow and can be differentiated by IL-3, IL-5, and GM-CSF [51]. And the prominent role of eosinophils in chronic colitis has been confirmed as GM-CSF regulation from Th17 cells [91]. GM-CSF secreted by Th17 cells maintains the eosinophilic mucosa and enables the activation of eosinophils [92]. In addition, previous studies found IL-23-Th17 cells feedback loop, wherein IL-23 maintained Th17 cell population, produced IL-17, and also induced Th17 cells to secrete GM-CSF, and GM-CSF in turn induced antigen-presenting cells to further secrete IL-23, thereby constantly maintaining the Th17 cell chronic reaction [93]. This means that, in eye allergies, Th17 may also aggravate the symptoms by this route. Therefore, IL-17 can promote the aggregation of IgE and eosinophils. This also further promotes the maintenance of the immune response. A 2017 study showed that IL-17A was involved in the pathophysiology of allergies by increasing the ability of IL-13 to activate signaling pathways such as intracellular signal transduction and activation of transcription factor 6 (STAT-6). This is the first mechanistic explanation of how IL-17A directly enhances Th2 response [91]. Study finds that IL-17 from T cells has a dose-dependent effect on IL-13-induced allergic airway inflammation [92]. So, higher doses of IL-17 can attenuate the inflammatory response induced by IL-13. There are also reports showing that increased IL-17A protein expression synergizes with IL-13 [68]. Laboratory has demonstrated that when IL-22 gene-knockout mice received induced airway eosinophils, IL-13 expression was reduced [62]. However, neutralization of IL-22 with an antibody increased IL-13 protein expression [94]. This means that IL-22 may have a dual role in allergies (Figure 3).

### 3.4. The Effects of Other Signaling Molecules on Th17 Cells

#### 3.4.1. IL-27

Recent studies have shown that IL-27 inhibits Th17 cell differentiation [52, 95, 96]. This also affected the Th2 response in mouse models of allergic conjunctivitis. Chen et al.'s research results [78] confirmed that the inhibition and depletion of the IL-27 signal intensified the dominant role of Th2, which was realized through reducing IL-27's antagonism of GATA3 expression [97]. They also confirmed that enhancing Th17 response by increasing ROR*γ*t exacerbated allergic inflammation [78]. At the same time, Th1 response was inhibited by suppression and consumption of the IL-27 signal, and the Th2 response advantage was further expanded. Their experiments also confirmed the promotion effect of Th17 on TH2 response.

#### 3.4.2. OPN

OPN expression is enhanced in Th2 diseases (nasal polyps and allergic rhinitis) in the Chinese population, suggesting that OPN may enhance Th2 response [98, 99]. Our study also provides possible evidence that OPN is involved in the Th17 response in AC. Several studies have investigated the role of OPN in promoting chemotactic inflammatory cells such as eosinophils and mast cells [100, 101]. The correlation between OPN and disease severity and high OPN expression during allergy season suggest that OPN can be used as a possible biomarker for the differential diagnosis of other diseases, monitoring disease activity or response to treatment [101].

## 4. Treatment and Management

### 4.1. Where Are We Now?

#### 4.1.1. Diagnosis

Diagnosis is based on allergic conjunctivitis clinical symptoms and conjunctival examination, but there are some laboratory tests that can usefully support this diagnosis [102, 103]. For example, skin tests for specific allergens can be performed by scratch tests or intradermal injections of allergens [104]. Some scholars have suggested skin prick tests should be included in the diagnostic work of AC patients for allergen immunotherapy [105]. These investigations should be able to find sensitivity to allergens including dust mites, animal dander, atmospheric mold, and seasonal pollen from grasses, trees, or weeds. Other scholars have suggested routine testing of food sensitivity in children, although food allergens are still controversial with regard to eye allergies [106]. Meanwhile, scraping the conjunctival surface to find eosinophils is a useful diagnostic method. The specific method is as follows: use the instrument to gently scrape several times on the inner surface of the conjunctiva. It is then stained with reagents. Check the slide for eosinophil granules or eosinophils. However, due to the presence of eosinophils in the conjunctiva typically deep, the upper layer may not be detected or not be eosinophils. Even the presence of only one eosinophil or eosinophil granule is important evidence for the diagnosis of allergic conjunctivitis, and the diagnosis of allergy should not be ruled out without eosinophils [28]. Vitro testing of IgE antibodies and specific allergens are widely used [27]. Some scholars claim that the results of IgE of tears and IgE of serum are sometimes inconsistent, so the IgE positive rate of tears may be more meaningful for local allergic conjunctivitis [107].

#### 4.1.2. Treatment and Management

Common treatments include eye drops containing antihistamine drugs, mast cell stabilizers, nonsteroidal drugs, and corticosteroids. Standard treatments are separate local antihistamine drug use or the use of local mast cell stabilizers alone or topical dual antihistamine-mast cell stabilizing agents [108–110]. They can effectively reduce the symptoms and signs of AC. Steroids can be given in the short term in the presence of severe symptoms and lack of response to other treatments [110]. Immunomodulators can effectively inhibit the activation of T cells and can treat severe allergic eye diseases. Immunomodulators alter the normal immune pathway and provide an alternative to steroids for allergic conjunctival disease [111]. Meanwhile, allergen immunotherapy is both safe and effective treatment [103]. In addition, the current major advances in treatment are immunotherapy, including classic subcutaneous and sublingual immunotherapy and novel subcutaneous and intralymphatic immunotherapy drug delivery systems, as well as edible rice vaccines [109, 112].

### 4.2. Future Diagnosis and Treatment Options

Th17 cells have been recently implicated in steroid resistance mechanisms. Recent evidence suggests that Th17 cells can have a dual response to glucocorticoids. According to immunopathology, they can be very sensitive to glucocorticoids or resistant to glucocorticoids, and this feature behavior has been stated in Banuelos et al.'s extensive overview [113]. Therefore, the tool-targeted IL-17 pathway may be more valuable for patients with hormone-resistant allergic conjunctivitis. For instance, common motif biomolecule of IL-17A and IL-17F is currently in clinical development, including nanobodies ALX-0761 and mAb bimekizumab [87, 114]. Moreover, IL-17A blocking antibodies sukinumab and ixekizumab have been recently used to treat psoriasis and ankylosing spondylitis. [115, 116]. Similarly, anti-IL-23 monoclonal therapy may be effective in eliminating the Th17 cell-eosinophil axis [51]. A simpler treatment may be used to inhibit eosinophil peroxidase with antioxidants such as vitamin E, limiting the main factors that cause the damage observed in these studies [51, 117]. Gallic acid treatment downregulated the expression of ROR*γ*t and IL-17 [71]. However, their effectiveness and safety in the application of allergic conjunctivitis are yet to be confirmed. However, their treatment of hormone-insensitive AC patients can provide more ideas.

## Abbreviations

AC: Allergic conjunctivitis
SAC: Seasonal allergic conjunctivitis
PAC: Perennial allergic conjunctivitis
AKC: Atopic keratoconjunctivitis
VKC: Vernal keratoconjunctivitis
GPC: Giant papillary conjunctivitis
Th: T-helper
IL: Interleukin
DC: Dendritic cells
APC: Antigen-presenting cells
MC: Mast cells
ROR*γ*t: Retinoid-related orphan receptor *γ*t
GM-CSF: Granulocyte-macrophage colony-stimulating factor
TGF-*β*: Transforming growth factor *β*
STAT3: Signal transduction and activation of transcription factor 3
STAT6: Signal transduction and activation of transcription factor 6.
